# Unraveling the connection: Inflammatory markers and diabetes mellitus pathogenesis

**DOI:** 10.1097/MD.0000000000047338

**Published:** 2026-01-23

**Authors:** Emmanuel Ifeanyi Obeagu

**Affiliations:** a Department of Biomedical and Laboratory Science, Africa University, Mutare, Zimbabwe.

**Keywords:** diabetes mellitus, inflammation, inflammatory markers, pathogenesis, therapeutic interventions

## Abstract

Diabetes mellitus is a multifaceted metabolic disorder characterized by chronic hyperglycemia, arising from defects in insulin secretion, insulin action, or both. Beyond its well-documented metabolic underpinnings, emerging evidence has illuminated a pivotal role of inflammation in the pathogenesis of diabetes. Pro-inflammatory cytokines, such as interleukin-6 (IL-6), tumor necrosis factor-alpha (TNF-α), and interleukin-1β (IL-1β), disrupt insulin signaling, impair β-cell function, and exacerbate insulin resistance. Chronic low-grade inflammation serves as a unifying mechanism linking obesity, metabolic dysfunction, and diabetes progression. The interplay between inflammatory pathways and diabetes extends to both type 1 and type 2 diabetes. In type 1 diabetes, autoimmune-mediated β-cell destruction is driven by inflammatory cytokines and dysregulated immune responses, while in type 2 diabetes, systemic and adipose tissue inflammation perpetuate insulin resistance and β-cell stress. Key molecular players, including toll-like receptors, the NLRP3 inflammasome, and the c-Jun N-terminal kinase (JNK) pathway, act as mediators between metabolic stress and inflammatory responses, emphasizing the bidirectional relationship between inflammation and hyperglycemia.

## 1. Introduction

The introduction to the complex interplay between inflammatory markers and the pathogenesis of diabetes mellitus (DM) unfolds a narrative deeply rooted in the modern understanding of metabolic disorders. DM, a chronic metabolic condition characterized by hyperglycemia resulting from defects in insulin secretion, insulin action, or both, poses a significant global health burden.^[1–3]^ Its prevalence has reached epidemic proportions, with approximately 463 million adults affected worldwide, a number projected to rise to 700 million by 2045.^[4]^ While traditional risk factors such as obesity, sedentary lifestyle, and genetic predisposition have long been recognized, emerging evidence highlights the pivotal role of inflammation in the pathophysiology of DM.^[5]^ Inflammation, once perceived solely as a defensive response to injury or infection, has garnered attention as a central player in the development and progression of DM.^[6]^ The concept of “metaflammation” - low-grade chronic inflammation associated with metabolic dysfunction - has gained prominence in recent years.^[7]^ This inflammatory state, characterized by elevated levels of cytokines, chemokines, and acute-phase reactants, disrupts insulin signaling pathways, promotes insulin resistance, and contributes to beta-cell dysfunction.^[8]^ Such disruptions collectively contribute to the pathogenesis of both type 1 and type 2 DM, blurring the lines between autoimmune-mediated destruction and metabolic dysregulation.

Central to the inflammatory milieu observed in DM is the dysregulated production of pro-inflammatory cytokines and adipokines.^[9]^ Adipose tissue, once viewed solely as an energy storage depot, is now recognized as an active endocrine organ secreting a myriad of bioactive molecules.^[10]^ Adipocytes, as well as resident immune cells within adipose tissue, produce inflammatory mediators such as tumor necrosis factor-alpha (TNF-α), interleukin-6 (IL-6), and adipokines such as leptin and adiponectin.^[11]^ Dysregulation of these adipose-derived factors disrupts metabolic homeostasis, contributing to insulin resistance, dyslipidemia, and systemic inflammation.Furthermore, emerging evidence suggests a bidirectional relationship between inflammation and insulin resistance, forming a vicious cycle that perpetuates metabolic dysfunction. Insulin resistance, characterized by impaired insulin action in target tissues such as liver, muscle, and adipose tissue, triggers compensatory hyperinsulinemia. Hyperinsulinemia, in turn, exacerbates inflammation by promoting the release of pro-inflammatory cytokines and reactive oxygen species.^[12]^ This bidirectional interplay amplifies the inflammatory response, further compromising insulin sensitivity and glucose homeostasis.

Moreover, the role of inflammatory markers extends beyond insulin resistance to encompass the pathogenesis of beta-cell dysfunction and apoptosis. Chronic exposure to pro-inflammatory cytokines such as interleukin-1 beta (IL-1β) and TNF-α induces endoplasmic reticulum stress, mitochondrial dysfunction, and oxidative stress in pancreatic beta-cells.^[13]^ These molecular derangements impair insulin secretion, exacerbate glucotoxicity and lipotoxicity, and ultimately contribute to beta-cell failure. Thus, inflammation emerges as a critical determinant of both insulin resistance and beta-cell dysfunction, driving the pathogenesis of DM through multifaceted mechanisms.The intricate relationship between inflammation and DM extends beyond the realm of pathophysiology to encompass clinical implications and therapeutic interventions. Elevated levels of inflammatory markers such as C-reactive protein (CRP), fibrinogen, and interleukin-6 (IL-6) have been associated with an increased risk of DM and its complications.^[14]^ These inflammatory biomarkers serve as prognostic indicators, aiding risk stratification and informing therapeutic decisions in clinical practice. Moreover, targeting inflammation through lifestyle modifications, pharmacotherapy, or anti-inflammatory agents holds promise in the management and prevention of DM. Lifestyle interventions such as weight loss, physical activity, and dietary modifications exert anti-inflammatory effects, improving insulin sensitivity and glycemic control. Pharmacological agents targeting specific inflammatory pathways, such as IL-1β antagonists and TNF-α inhibitors, have shown efficacy in improving glycemic parameters and reducing cardiovascular risk in individuals with DM.^[14]^

### 1.1. Aim

The aim of this study is to comprehensively investigate the intricate relationship between inflammatory markers and the pathogenesis of DM.

### 1.2. Rationale

The rationale behind this study stems from the growing recognition of inflammation as a key contributor to the pathogenesis of DM. While traditional risk factors such as obesity and sedentary lifestyle have long been associated with diabetes, emerging evidence suggests that chronic low-grade inflammation plays a pivotal role in driving metabolic dysfunction and insulin resistance, hallmarks of DM. Moreover, despite the established association between inflammation and DM, several key questions remain unanswered. These include the specific inflammatory pathways involved, the temporal relationship between inflammation and metabolic dysfunction, and the clinical implications of elevated inflammatory markers in individuals with DM. By addressing these gaps in knowledge, this study aims to provide a deeper understanding of the complex interplay between inflammation and DM, laying the groundwork for more effective diagnostic and therapeutic strategies. Furthermore, elucidating the clinical implications of elevated inflammatory markers in individuals with DM is of paramount importance for risk stratification and therapeutic decision-making in clinical practice. By investigating the prognostic significance of inflammatory markers and their utility as therapeutic targets, this study aims to inform personalized treatment approaches that address the underlying inflammatory processes driving DM.

## 2. Review methodology

### 2.1. Literature search strategy

A systematic literature search was conducted using electronic databases such as PubMed, Scopus, and Web of Science. The search strategy included a combination of keywords related to inflammation (e.g., “inflammatory markers,” “cytokines,” “adipokines”) and DM (e.g., “type 1 diabetes,” “type 2 diabetes,” “insulin resistance”). Boolean operators (AND, OR) were utilized to refine the search results and capture relevant studies.

### 2.2. Inclusion and exclusion criteria

Studies were included based on predefined criteria, including relevance to the topic, publication in peer-reviewed journals, and availability of full-text articles in English. Both experimental studies (e.g., animal models, in vitro experiments) and clinical studies (e.g., cohort studies, clinical trials) were considered. Studies focusing on other metabolic disorders or unrelated to inflammatory markers and DM were excluded.

### 2.3. Screening and selection process

Titles and abstracts of the identified articles were screened independently by 2 reviewers to determine eligibility for inclusion. Full-text articles of potentially relevant studies were then assessed to confirm eligibility. Any discrepancies between reviewers were resolved through discussion and consensus.

### 2.4. Quality assessment

The quality of included studies was evaluated using appropriate quality assessment tools tailored to the study design (e.g., Newcastle-Ottawa Scale for cohort studies, Cochrane Risk of Bias Tool for clinical trials). Quality assessment was conducted independently by 2 reviewers, and any discrepancies were resolved through discussion.

### 2.5. Critical appraisal and interpretation

The synthesized findings were critically appraised to assess the strength of evidence, identify gaps in knowledge, and draw conclusions regarding the role of inflammatory markers in DM pathogenesis. The implications of the findings for clinical practice and future research directions were also discussed.

### 2.6. Ethical approval

Not applicable.

### 2.7. Inflammatory markers in diabetes mellitus

Inflammatory markers in DM are substances that are associated with inflammation and are often elevated in individuals with diabetes. Inflammation is a complex biological response to harmful stimuli and plays a significant role in the pathogenesis, progression, and complications of diabetes. C-reactive protein (CRP) is a well-established acute-phase protein produced by the liver in response to inflammation. Elevated CRP levels are associated with insulin resistance and are a risk factor for type 2 diabetes (T2D) and its complications.Various interleukins, such as IL-1, IL-6, and IL-18, are pro-inflammatory cytokines implicated in the inflammatory processes of diabetes. IL-6, in particular, is associated with insulin resistance and T2D.Tumor Necrosis Factor-alpha (TNF-α) is a cytokine that contributes to insulin resistance by interfering with insulin signaling. Increased TNF-α levels are observed in obesity and T2D.Adiponectin is an anti-inflammatory adipokine that has protective effects in diabetes. Low levels of adiponectin are associated with insulin resistance and an increased risk of T2D.Leptin is another adipokine, and its elevated levels in obesity can contribute to inflammation and insulin resistance.Monocyte Chemoattractant Protein-1 (MCP-1) is a chemokine that plays a role in recruiting monocytes to sites of inflammation. It has been linked to inflammation in diabetes and the development of diabetic complications.^[14–20]^

These inflammatory markers are not only indicative of the presence of inflammation in diabetes but can also serve as diagnostic and prognostic tools.^[21]^ For example, HbA1c, a marker used for monitoring long-term blood glucose control, has been associated with inflammation, and its levels may be influenced by inflammatory processes.^[22]^ Emerging biomarkers and advanced imaging techniques are also being explored for their potential to provide insight into the inflammatory component of diabetes.In addition to their diagnostic significance, these markers offer potential therapeutic implications. Strategies to reduce inflammation, such as lifestyle interventions, anti-inflammatory drugs, and emerging therapies, are being investigated as approaches to mitigate the impact of inflammation on diabetes management and its associated complications.

### 2.8. Mechanisms of inflammation in diabetes

The mechanisms of inflammation in diabetes are complex and multifaceted, involving a variety of pathways and cellular interactions.^[23]^ Inflammation plays a pivotal role in the pathogenesis, progression, and complications of diabetes, particularly in type 2 diabetes (T2D). In individuals with obesity and insulin resistance, there is often a state of chronic low-grade inflammation. Adipose tissue, particularly visceral fat, serves as a significant source of pro-inflammatory molecules.^[12]^ This chronic inflammation is characterized by elevated levels of pro-inflammatory cytokines such as tumor necrosis factor-alpha (TNF-α) and interleukin-6 (IL-6).Inflammation in diabetes is closely linked to immune system dysregulation. Macrophages, a type of white blood cell, infiltrate adipose tissue and become activated, producing pro-inflammatory cytokines.^[14]^ This immune response contributes to insulin resistance by interfering with insulin signaling in target tissues.Adipose tissue acts as an endocrine organ, secreting adipokines that influence metabolism and inflammation. In obese individuals, adipose tissue becomes dysfunctional and releases more pro-inflammatory adipokines, such as leptin and less anti-inflammatory adipokines like adiponectin. This imbalance contributes to systemic inflammation.

Inflammation can lead to endothelial dysfunction, characterized by impaired function of the blood vessel lining. This dysfunction reduces nitric oxide production and increases the release of pro-inflammatory factors, contributing to insulin resistance and cardiovascular complications in diabetes.^[24]^ Chronic inflammation is associated with oxidative stress, which involves an imbalance between the production of reactive oxygen species and the body’s ability to neutralize them. Oxidative stress can lead to damage of cells, tissues, and DNA, exacerbating insulin resistance and contributing to beta-cell dysfunction in the pancreas.^[25]^ In type 1 diabetes (T1D), inflammation occurs in the pancreas itself, particularly in the islets of Langerhans.^[26]^ Immune cells infiltrate these islets and attack insulin-producing beta-cells, leading to their destruction.^[27]^ This autoimmune response is a hallmark of T1D.Emerging research suggests that the gut microbiota plays a role in diabetes-related inflammation. An imbalance in the gut microbiome, known as dysbiosis, can lead to increased gut permeability, allowing pro-inflammatory bacterial products to enter the bloodstream and trigger systemic inflammation.High blood glucose levels can lead to the formation of advanced glycation end products, which can stimulate inflammation by binding to receptors on immune cells and promoting the release of pro-inflammatory cytokines.

### 2.9. Inflammatory markers as diagnostic tools in diabetes mellitus

Inflammatory markers are increasingly recognized as valuable diagnostic tools in DM. They provide insights into the presence and extent of inflammation associated with the condition. Utilizing these markers in the diagnostic process can help in assessing the risk of diabetes, monitoring disease progression, and guiding treatment strategies. Elevated C-Reactive Protein (CRP) levels are indicative of systemic inflammation and are associated with insulin resistance and the development of type 2 diabetes (T2D). Measuring CRP can be useful in identifying individuals at risk of T2D and assessing their inflammatory status.^[13]^ Pro-inflammatory cytokines like IL-6 have been linked to insulin resistance and T2D. Measuring these cytokines can provide insights into the level of inflammation in individuals with diabetes and help in monitoring the effectiveness of interventions.Tumor Necrosis Factor-alpha (TNF-α) is a key cytokine associated with insulin resistance. Elevated levels of TNF-α may serve as an early warning sign of developing T2D and can be used for risk assessment.^[14]^

Measuring adipokines, such as adiponectin and leptin, provides information about adipose tissue inflammation and its impact on metabolic health. Low adiponectin and high leptin levels are often observed in individuals with obesity and T2D.^[12]^ Monocyte Chemoattractant Protein-1 (MCP-1) is a chemokine that plays a role in recruiting immune cells to sites of inflammation. Elevated levels of MCP-1 are observed in individuals with diabetes and can be used to assess the extent of inflammation.While primarily a marker of long-term blood glucose control, HbA1c levels can also be influenced by inflammation. High levels of inflammation can lead to higher HbA1c values, potentially suggesting the presence of underlying inflammation in diabetes.^[28]^ These markers may include high-sensitivity CRP (hs-CRP), fetuin-A, and others.^[29]^ Advanced imaging techniques, such as positron emission tomography (PET) and magnetic resonance imaging (MRI), are being explored to visualize inflammation in various tissues, including adipose tissue and the pancreas. These techniques can aid in understanding the distribution and extent of inflammation in diabetes.^[29]^

### 2.10. Inflammatory markers in diabetes-related complications

Chronic inflammation is a central player in the pathogenesis of cardiovascular disease, and it is closely associated with diabetes. Inflammatory markers like C-reactive protein (CRP), interleukin-6 (IL-6), and tumor necrosis factor-alpha (TNF-α) are elevated in individuals with diabetes and contribute to atherosclerosis, endothelial dysfunction, and increased cardiovascular disease (CVD) risk. Inflammation is involved in the development and progression of diabetic nephropathy, a common complication of diabetes.^[14]^ Elevated levels of MCP-1 (monocyte chemoattractant protein-1) and other inflammatory markers are associated with renal inflammation and fibrosis, leading to kidney damage.Inflammatory markers contribute to the pathogenesis of diabetic retinopathy, a leading cause of blindness in individuals with diabetes.^[30]^ Factors such as vascular endothelial growth factor and inflammatory cytokines play a role in the development of retinal vascular abnormalities and leakage.Inflammation and oxidative stress are implicated in the development of diabetic neuropathy, which can result in nerve damage. Elevated levels of inflammatory markers are observed in nerve tissues and may contribute to nerve dysfunction.^[30,31]^

Individuals with diabetes are at an increased risk of nonalcoholic fatty liver disease (NAFLD), a condition characterized by liver inflammation and fat accumulation.^[31]^ Inflammatory markers, including CRP and TNF-α, are often elevated in individuals with NAFLD associated with diabetes.Diabetes is a significant risk factor for coronary artery disease, and inflammation is central to its pathogenesis.^[31]^ Inflammatory markers are associated with the development of atherosclerotic plaques and can lead to coronary artery disease. Peripheral arterial disease is another vascular complication of diabetes. Inflammation contributes to the atherosclerotic process in peripheral arteries, resulting in reduced blood flow.^[32]^ Markers such as CRP and IL-6 are linked to peripheral arterial disease in individuals with diabetes.While not a direct complication, oxidative stress resulting from inflammation is a common denominator in many diabetes-related complications. It can lead to cellular damage and contribute to complications in various organs and systems.

### 2.11. Therapeutic implications of inflammatory markers in diabetes mellitus

While diabetes management traditionally focuses on blood glucose control, addressing inflammation is becoming increasingly important. Exercise helps reduce pro-inflammatory markers like CRP and IL-6 while improving insulin sensitivity.^[14]^ A diet rich in anti-inflammatory foods, such as fruits, vegetables, whole grains, and omega-3 fatty acids, can help mitigate inflammation.Obesity is closely associated with chronic inflammation and insulin resistance.^[33]^ Weight loss, achieved through a combination of calorie restriction and increased physical activity, can significantly reduce inflammation and improve glycemic control in individuals with diabetes. Anti-inflammatory drugs may be considered in some cases to target inflammation in diabetes.^[34]^ For example, nonsteroidal anti-inflammatory drugs (NSAIDs) or specific anti-inflammatory agents may be prescribed to individuals with diabetes and significant inflammation, but these should be used with caution and under medical supervision.

Metformin, a commonly used medication in diabetes management, has shown potential anti-inflammatory properties.^[35]^ It may help reduce inflammation and improve insulin sensitivity in addition to its glucose-lowering effects. Antioxidants, such as vitamin C and vitamin E, can help counteract oxidative stress and inflammation associated with diabetes.^[36]^ These antioxidants may be included in the treatment regimen of individuals with diabetes, although their benefits are still a subject of ongoing research. Emerging therapies are being developed to specifically target inflammation in diabetes. These may include anti-inflammatory monoclonal antibodies or small molecule inhibitors of pro-inflammatory pathways. In type 1 diabetes (T1D), where the autoimmune response plays a central role, immunomodulatory therapies may be used to regulate the immune system and reduce inflammation in the pancreas. These therapies aim to slow or halt the destruction of insulin-producing beta-cells. Personalized treatment approaches, tailored to the individual’s inflammatory profile, and are increasingly considered. Identifying specific inflammatory markers that are elevated in a patient can guide treatment decisions. Some individuals may benefit from anti-inflammatory interventions more than others.Regular monitoring of inflammatory markers can help healthcare providers assess the effectiveness of treatment and the risk of complications. High levels of inflammation may warrant more aggressive intervention or closer monitoring.^[14]^ Table 1 shows diagnostic biomarkers in DM, Table 2 shows prognostic biomarkers in DM, Table 3 shows risk factors associated with DM and inflammation, Table 4 shows diagnostic value of inflammatory markers in DM and Table 5 shows therapeutic targets and inflammatory markers in DM (provided by author).

**Table 1:** Diagnostic biomarkers in DM.

Biomarker	Type of diabetes	Detection method	Reported thresholds	Clinical relevance
IL-1β	T1DM, T2DM	ELISA, Multiplex	>5 pg/mL	Early detection of β-cell dysfunction
IL-6	T2DM	ELISA, Multiplex	>10 pg/mL	Indicator of insulin resistance and inflammation
TNF-α	T2DM	ELISA	>15 pg/mL	Associated with systemic inflammation and insulin resistance
CRP	T1DM, T2DM	High-sensitivity ELISA	>3 mg/L	Marker of chronic low-grade inflammation
MCP-1 (CCL2)	T2DM	ELISA	>50 pg/mL	Reflects adipose tissue inflammation

CRP = C-reactive protein, DM = diabetes mellitus, IL-1β = interleukin-1 beta, IL-6 = interleukin-6, MCP-1 = monocyte chemoattractant protein-1, TNF-α = tumor necrosis factor-alpha.

**Table 2:** Prognostic biomarkers in DM.

Biomarker	Type of diabetes	Detection method	Prognostic threshold	Associated outcome
IL-18	T2DM	ELISA	>300 pg/mL	Predicts progression from prediabetes to T2DM
IL-10	T2DM	ELISA	<10 pg/mL	Low levels associated with poor glycemic control
TNF-α	T2DM	ELISA	>15 pg/mL	Predicts cardiovascular complications
CRP	T1DM, T2DM	High-sensitivity ELISA	>5 mg/L	Correlates with micro- and macrovascular complications
Adiponectin	T2DM	ELISA	<7 μg/mL	Low levels predict insulin resistance progression

CRP = C-reactive protein, DM = diabetes mellitus, TNF-α = tumor necrosis factor-alpha.

**Table 3:** Risk factors associated with DM and inflammation.

Risk factor	Associated biomarkers	Mechanistic insight
Obesity	IL-6, TNF-α, MCP-1	Adipose tissue inflammation drives insulin resistance
Sedentary lifestyle	IL-6, CRP	Physical inactivity promotes systemic inflammation
Age	IL-6, TNF-α	Aging-associated immune dysregulation increases diabetes risk
Genetic predisposition	IL-1β, IL-18	Cytokine gene polymorphisms influence inflammatory response
High-fat diet	TNF-α, MCP-1	Diet-induced adipose inflammation contributes to insulin resistance

CRP = C-reactive protein, DM = diabetes mellitus, IL-1β = interleukin-1 beta, IL-6 = interleukin-6, MCP-1 = monocyte chemoattractant protein-1, TNF-α = tumor necrosis factor-alpha.

**Table 4:** Diagnostic value of inflammatory markers in DM.

Diagnostic marker	Diagnostic value
High-sensitivity CRP (hs-CRP)	Predicts risk of diabetes mellitus
IL-6	Correlated with insulin resistance, diabetes mellitus severity
MCP-1	Associated with early stages of diabetes mellitus

CRP = C-reactive protein, DM = diabetes mellitus, IL-6 = interleukin-6, MCP-1 = monocyte chemoattractant protein-1.

**Table 5:** Therapeutic targets and inflammatory markers in DM.

Therapeutic target	Impact on inflammatory marker
Exercise	Reduces CRP, IL-6 levels
Anti-inflammatory drugs	Lower levels of TNF-α, IL-1β
Diet modification	Decreases inflammatory marker expression

CRP = C-reactive protein, DM = diabetes mellitus, IL-1β = interleukin-1 beta, IL-6 = interleukin-6, TNF-α = tumor necrosis factor-alpha..

### 2.12. Metabolic pathways

Metabolic pathways are interconnected series of chemical reactions occurring within cells that are essential for maintaining life. These pathways facilitate the conversion of molecules into energy and the synthesis of biomolecules necessary for growth, maintenance, and reproduction. Glycolysis is the initial pathway of glucose metabolism, occurring in the cytoplasm of cells. It breaks down glucose (a 6-carbon sugar) into 2 molecules of pyruvate (a 3-carbon compound), generating ATP and NADH in the process. It provides energy for cellular processes and intermediates for other metabolic pathways, such as the citric acid cycle and gluconeogenesis. The citric acid cycle takes place in the mitochondria and completes the oxidation of glucose-derived pyruvate to carbon dioxide. It produces ATP, NADH, and FADH₂, which carry high-energy electrons to the electron transport chain. It generates a significant amount of ATP and supplies intermediates for biosynthesis.^[33]^

Located in the inner mitochondrial membrane, the electron transport chain uses the electrons carried by NADH and FADH₂ from glycolysis and the citric acid cycle to generate ATP through oxidative phosphorylation. It is the major ATP-generating pathway in aerobic organisms, producing most of the cell’s ATP. Gluconeogenesis is the process of synthesizing glucose from noncarbohydrate precursors (such as lactate, glycerol, and amino acids), mainly occurring in the liver and to a lesser extent in the kidneys. It maintains blood glucose levels during fasting or starvation and provides glucose for tissues that cannot use fatty acids as an energy source (e.g., red blood cells and brain). Glycogenesis is the formation of glycogen (a storage form of glucose) from glucose molecules. It occurs in the liver and muscle cells when blood glucose levels are high. Glycogenolysis is the breakdown of glycogen to glucose, providing glucose for energy production when blood glucose levels are low, such as during fasting or exercise. Lipid metabolism involves the synthesis (lipogenesis) and breakdown (lipolysis) of fats (triglycerides), which are important for energy storage and structural components of cell membranes. It provides a source of energy (fatty acids and ketone bodies) and contributes to membrane structure, hormone synthesis, and insulation. Protein metabolism includes protein synthesis (anabolism) and breakdown (catabolism), regulating cellular function, growth, repair, and maintenance. Amino acids from protein breakdown can be used for energy production or as precursors for nucleotide and hormone synthesis. The pentose phosphate pathway is an alternative pathway of glucose metabolism that generates NADPH (used in biosynthetic reactions and antioxidant defenses) and ribose-5-phosphate (for nucleotide synthesis). It supports biosynthetic processes (e.g., nucleotide synthesis) and provides reducing equivalents (NADPH) for anabolic reactions and detoxification. Metabolic pathways are tightly regulated by enzymes, hormones, and cellular energy status (e.g., ATP/ADP ratio). Regulation ensures that energy production and biomolecule synthesis meet the cell’s needs while responding to external factors like nutrient availability and stress.^[34]^

### 2.13. Advanced researches

Advanced research in metabolic pathways encompasses a wide array of interdisciplinary studies that delve deep into the molecular mechanisms, regulatory networks, and clinical implications of metabolic processes. Metabolomics is the comprehensive study of small molecules (metabolites) involved in cellular processes within a biological system. It provides insights into metabolic pathways, metabolic fluxes, and biomarker discovery. Recent advancements include high-resolution mass spectrometry and nuclear magnetic resonance spectroscopy for metabolite profiling, which enable detailed analysis of metabolic changes in health and disease states. Systems biology integrates computational, mathematical, and experimental methods to study complex biological systems, including metabolic pathways. Systems biology models metabolic networks to predict system-wide behaviors, understand metabolic fluxes, and identify key nodes for therapeutic intervention. It involves quantitative analysis of omics data (genomics, transcriptomics, proteomics, metabolomics) to reconstruct metabolic networks and simulate metabolic responses under different conditions. Mitochondria are organelles central to cellular metabolism, generating ATP through oxidative phosphorylation and regulating metabolic signaling pathways. Current research focuses on mitochondrial dynamics, bioenergetics, and their role in metabolic diseases. Studies explore mitochondrial dysfunction in aging, neurodegenerative disorders, cancer metabolism, and metabolic syndrome, aiming to develop targeted therapies.^[33]^

Metabolic pathways are tightly regulated by signaling networks, including hormones, growth factors, and nutrient-sensing pathways (e.g., insulin signaling, AMPK pathway). Advances include understanding how signaling pathways integrate with metabolic networks to regulate energy balance, nutrient utilization, and cellular homeostasis. Research also explores metabolic reprogramming in cancer cells and metabolic adaptations in response to stress and environmental cues. Cells and organisms sense and respond to changes in nutrient availability through intricate signaling pathways. Recent studies focus on nutrient-sensing mechanisms (e.g., mTOR pathway, SIRT1/AMPK pathways) and metabolic adaptations during fasting, exercise, and dietary interventions. These insights have implications for metabolic health, longevity, and disease prevention. Metabolic engineering aims to manipulate metabolic pathways in organisms for biotechnological applications, such as biofuel production, pharmaceutical synthesis, and bioremediation. Recent advances include using synthetic biology tools (e.g., CRISPR/Cas9) for precise genome editing to optimize metabolic pathways in microbial hosts. Researchers are also developing novel biosensors and metabolic switches for controlling metabolic fluxes and enhancing production yields in industrial biotechnology. Metabolic research translates findings into clinical applications for diagnosing, treating, and preventing metabolic diseases.^[34]^

### 2.14. Challenges and future directions of inflammatory markers in diabetes mellitus

Challenges and future directions in the use of inflammatory markers in DM represent areas where research and clinical practice are evolving to improve the management of diabetes and its associated complications.

#### 2.14.1. Challenges

One of the challenges is the lack of standardized assays for measuring inflammatory markers.^[37]^ Variability in laboratory methods and reference ranges can make it difficult to compare results between studies and establish uniform diagnostic criteria.^[38]^ People with diabetes can exhibit significant interindividual variability in their inflammatory profiles. This variability complicates treatment decisions, as the same therapeutic approach may not be effective for all individuals with diabetes.The timing of when inflammatory markers are assessed can affect the results. It is essential to determine the most relevant time points for assessing inflammation in relation to diabetes progression and complications.Diabetes is a heterogeneous condition with different subtypes and degrees of inflammation. Tailoring treatments to individual inflammatory profiles can be challenging.While the role of inflammation in diabetes is recognized, there is a limited range of approved anti-inflammatory therapies for diabetes management. Expanding treatment options and determining which individuals will benefit most from them is a challenge.^[39–41]^

#### 2.14.2. Future directions

The future of diabetes care likely involves a personalized medicine approach that considers an individual’s unique inflammatory profile.^[42]^ Personalized treatment plans may involve the use of specific anti-inflammatory agents based on a patient’s inflammatory markers.The development of novel inflammatory markers and more precise measurement techniques can provide a more comprehensive assessment of inflammation in diabetes.^[43]^ These markers may offer better predictive value for complications.The integration of inflammatory marker assessments into routine diabetes care can help in early risk stratification and more tailored treatment approaches.^[44]^ An interdisciplinary care model, with collaboration between endocrinologists, rheumatologists, immunologists, and other specialists, can be beneficial.^[14]^ Advanced imaging techniques, such as PET and MRI, are being explored to visualize inflammation in various tissues, including adipose tissue and the pancreas.^[45]^ These techniques can help in understanding the distribution and extent of inflammation.Lifestyle interventions, such as diet and exercise, are likely to remain central in managing inflammation in diabetes. Research on dietary patterns and physical activity in reducing inflammation will continue to advance. Conducting well-designed clinical trials to assess the efficacy of anti-inflammatory therapies and interventions in diabetes management is essential.^[45]^ These trials can help determine which treatments are most effective in specific patient populations. Utilizing telemedicine and digital health technologies for remote monitoring and management of diabetes, including the assessment of inflammatory markers, can improve access to care and long-term follow-up.^[43–46]^

## 3. Conclusion

Inflammation is a central component in the pathogenesis of both type 1 and type 2 DM, influencing β-cell function, insulin resistance, and the development of vascular complications. Key inflammatory markers, including IL-1β, IL-6, TNF-α, CRP, and adipokines, provide valuable insights into disease mechanisms and hold promise as diagnostic, prognostic, and risk-stratification tools. Despite growing evidence, important controversies remain regarding the causal versus associative roles of specific cytokines, the reliability and standardization of biomarker measurements across populations and assay platforms, and the clinical efficacy of anti-inflammatory interventions. These uncertainties underscore the need for longitudinal studies, standardized assays, and rigorous clinical trials to validate biomarkers and therapeutic strategies. Integrating inflammatory markers into clinical practice offers the potential for early detection, personalized risk assessment, and tailored interventions, moving toward precision medicine in diabetes management. Future research addressing current gaps and controversies will be critical to fully harness the translational potential of these biomarkers and improve patient outcomes.

## Author contributions

**Conceptualization:** Emmanuel Ifeanyi Obeagu.

**Methodology:** Emmanuel Ifeanyi Obeagu.

**Resources:** Emmanuel Ifeanyi Obeagu.

**Supervision:** Emmanuel Ifeanyi Obeagu.

**Validation:** Emmanuel Ifeanyi Obeagu.

**Visualization:** Emmanuel Ifeanyi Obeagu.

**Writing – original draft:** Emmanuel Ifeanyi Obeagu.

**Writing – review & editing:** Emmanuel Ifeanyi Obeagu.
